# Multimorbidity among Diverse Communities in New England: Findings from the Healthy Aging Data Reports

**DOI:** 10.1093/geroni/igab046.2390

**Published:** 2021-12-17

**Authors:** Maki Karakida, Chae Man Lee, Taylor Jansen, Shu Xu, Frank Porell, Nina Silverstein, Elizabeth Dugan

**Affiliations:** 1 UMass Boston, Boston, Massachusetts, United States; 2 University of Masschusetts Boston, Dorchester, Massachusetts, United States; 3 University of Massachusetts Boston, University of Massachusetts Boston, Massachusetts, United States; 4 University of Massachusetts Boston, Boston, Massachusetts, United States

## Abstract

The risk for multimorbidity increases with age. Community burden of comorbidities in New England (NE) was assessed by comparing state and community rates of two measures (having no comorbidities and having 4 or more) among Medicare beneficiaries age 65+ in CT, MA, NH, and RI. Data sources were the Medicare Current Beneficiary Summary File (2014-2017) and the American Community Survey (2014-2018). Small area estimation techniques were used to calculate age-sex adjusted community rates. Multimorbidity was measured as people with zero or with 4 or more of the following chronic conditions: Alzheimer’s disease, asthma, atrial fibrillation, cancer (breast, colorectal, lung, and prostate), kidney disease, COPD, depression, diabetes, congestive heart failure, hypertension, hyperlipidemia, ischemic heart disease, osteoporosis, arthritis, and stroke. Rates for 4+ conditions: RI 63.8% (45.76-70.69%), CT 61.8% (47.82-70.05%), MA 60.7% (40-74.96%), NH 54.4% (36.67-62.99%). Results were mapped, showing the statewide and regional distribution of rates. Rates were much higher for having 4+ chronic conditions than not having any comorbidities. RI had the highest rates of 4+ and in MA the highest chronic disease rates were found in lower socioeconomic communities. CT has the highest number of diverse older residents and dual-eligible beneficiaries for Medicare and Medicaid in NE. The rates show late-life health disparities that have implications for independent living, quality of life, and mortality suggesting the need for policies to provide equitable access to care and resources to disadvantaged NE communities.

